# Plasticity and genetic variation in traits underpinning asexual replication of the rodent malaria parasite, *Plasmodium chabaudi*

**DOI:** 10.1186/s12936-019-2857-0

**Published:** 2019-07-01

**Authors:** Philip L. G. Birget, Kimberley F. Prior, Nicholas J. Savill, Lewis Steer, Sarah E. Reece

**Affiliations:** 1 Institute of Evolutionary Biology, School of Biological Sciences, University of Edinburgh, Charlotte Auerbach Road, Edinburgh, EH9 3FL UK; 2 Institute of Immunology and Infection Research, School of Biological Sciences, University of Edinburgh, Charlotte Auerbach Road, Edinburgh, EH9 3FL UK

**Keywords:** Red blood cell preference, Cycle duration, Phenotypic plasticity, In-host survival, Anaemia, G×E, Genotype by environment, Life history trait, Virulence

## Abstract

**Background:**

The ability of malaria (*Plasmodium*) parasites to adjust investment into sexual transmission stages versus asexually replicating stages is well known, but plasticity in other traits underpinning the replication rate of asexual stages in the blood has received less attention. Such traits include burst size (the number of merozoites produced per schizont), the duration of the asexual cycle, and invasion preference for different ages of red blood cell (RBC).

**Methods:**

Here, plasticity [environment (E) effects] and genetic variation [genotype (G) effects] in traits relating to asexual replication rate are examined for 4 genotypes of the rodent malaria parasite *Plasmodium chabaudi*. An experiment tested whether asexual dynamics differ between parasites infecting control versus anaemic hosts, and whether variation in replication rate can be explained by differences in burst size, asexual cycle, and invasion rates.

**Results:**

The within-host environment affected each trait to different extents but generally had similar impacts across genotypes. The dynamics of asexual densities exhibited a genotype by environment effect (G×E), in which one of the genotypes increased replication rate more than the others in anaemic hosts. Burst size and cycle duration varied between the genotypes (G), while burst size increased and cycle duration became longer in anaemic hosts (E). Variation in invasion rates of differently aged RBCs was not explained by environmental or genetic effects. Plasticity in burst size and genotype are the only traits making significant contributions to the increase in asexual densities observed in anaemic hosts, together explaining 46.4% of the variation in replication rate.

**Conclusions:**

That host anaemia induces several species of malaria parasites to alter conversion rate is well documented. Here, previously unknown plasticity in other traits underpinning asexual replication is revealed. These findings contribute to mounting evidence that malaria parasites deploy a suite of sophisticated strategies to maximize fitness by coping with, or exploiting the opportunities provided by, the variable within-host conditions experienced during infections. That genetic variation and genotype by environment interactions also shape these traits highlights their evolutionary potential. Asexual replication rate is a major determinant of virulence and so, understanding the evolution of virulence requires knowledge of the ecological (within-host environment) and genetic drivers of variation among parasites.

**Electronic supplementary material:**

The online version of this article (10.1186/s12936-019-2857-0) contains supplementary material, which is available to authorized users.

## Background

All parasites are adapted to extract energy from their host and divert it towards their own survival and reproduction. To survive within the host, malaria (*Plasmodium*) parasites must first replicate asexually in the liver, and then in the blood, [1]. Transmission (i.e., reproduction) requires the production of sexual stages (gametocytes) in the host, which mate when taken up by an insect vector. In the host’s blood, the vast majority of parasite biomass consists of asexually replicating stages, adapted for foraging on host resources and serving as a source population for the production of gametocytes [2]. Parasite traits that underpin asexual replication rate include burst size (the number of merozoites produced by a schizont [3]), preferential invasion of red blood cells (RBCs) of certain ages, the duration of the asexual cycle, the ability of parasites to avoid clearance (e.g., by the spleen) through sequestration [4], and the conversion rate [5, 6].

The conversion rate is well studied in *Plasmodium chabaudi* [7–9] and so the focus here is on other traits (hereafter termed ‘asexual traits’) affecting asexual replication, namely: burst size, the duration of the asexual cycle, and invasion rates of mature (normocytes) and immature (reticulocytes) RBCs. Variation in asexual traits has been observed within and between different species of *Plasmodium* [10–13]. Antia et al. [12], for example suggest that different genotypes of *P. chabaudi* have different age preferences for the RBCs they infect, and genetic variation in invasion preference, burst sizes and parasite effects on host erythropoiesis are thought to contribute to virulence differences between *P*. *chabaudi* genotypes [3, 11]. In addition to ‘genetically hardwired’ variation, asexual traits of a given genotype can vary according to changes in the within-host environment (‘phenotypic plasticity’). Furthermore, different parasite genotypes may react differently to the same change in within-host conditions (‘genotype by environment effects’). For example, the densities of asexuals and gametocytes produced by several *P. chabaudi* genotypes are differentially affected by host strain [14], and burst size is predicted to be higher if parasites develop inside reticulocytes compared to normocytes, especially for more virulent genotypes [3]. Environmentally driven variation in traits of the human malaria parasite *Plasmodium falciparum* are difficult to assess in natural infections due to the need to study parasites in culture, whereas examples of genetic variation are plentiful [15]: RBC invasion phenotypes [16], *var* gene repertoire [17] and anti-malarial drug resistance mutations [18].

All else being equal, simple predictions to act as null hypotheses can be made for how asexual traits affect replication rate. For example, a high burst size should result in rapid increase in asexual density. If parasites can alter their ability to invade mature and immature RBCs to match the changing age structure of RBCs during infection, as hosts develop and then recover from anaemia, they could maximize replication rate throughout infections. Burst size may also be plastic in response to the age of host RBC and drive variation in cycle-to-cycle replication during infections [3, 11]. In contrast, a faster asexual cycle may not lead to fast replication because development during asexual replication coordinates with host circadian rhythms [19–21], so (stabilizing) selection may favour parasites with a cycle closest to the 24-h duration of host rhythms. Finally, because each individual parasite can develop either as an asexual or a gametocyte, a resource allocation trade-off exists in which the more parasites convert to gametocytes, the slower the asexual replication rate [5]. Similar to the trade-off involved in conversion, trade-offs may govern interactions between other traits. A high burst size might come at a cost of elongating cycle duration, or merozoites must specialize on the expression of ligands for invading RBC of certain ages [22].

Genetic variation, plasticity and genotype by environment effects are important to quantify because they may maintain genetic variation in populations as well as allowing genetic variation to be exposed to natural selection [23–25]. However, these concepts are hard to study for parasites due to the difficulty of separating parasite control of traits from variation that is caused by the direct impact of environmental change (i.e., host control of parasite traits). For example, host control of immune responses and RBC resources also mediates how permissive the within-host environment is to replication [12, 13, 26, 27]. However, there is evidence that parasites are at least in part responsible for adjusting asexual traits. For example, [10] show that burst size is reduced in calorie-restricted hosts and this is not simply because parasites fail to acquire sufficient resources for maximum merozoite production, but instead, that they appear to use a nutrient sensing pathway to match burst size to the resources available.

This study investigates genetic variation, phenotypic plasticity and genotype by environment interactions in replication rate and the asexual traits that underpin replication rate. An experiment was conducted to test whether the dynamics of asexual densities and the traits that underpin replication (burst size, asexual cycle duration and RBC invasion preference) vary between parasites infecting anaemic and control hosts, for 4 genotypes of *P. chabaudi*. Sequestration of asexual stages was not considered due to the difficulties of obtaining reliable estimates. Finally, the relative contributions of asexual traits to the variation observed in replication rate were assessed. The findings reveal genetic variation (G), plasticity (E) and genotype by environment interactions (G×E) in asexual traits and suggest that plasticity in burst size is the main contributor to faster replication in anaemic hosts. Quantifying the genetic variation, plasticity and genotype by environment interactions involved in asexual traits, and how variation in traits acts in concert, may reveal new intervention opportunities where targeting one trait may affect other traits through trade-offs or pleiotropy in clinically favourable ways [28].

## Methods

### Experimental design

Fifty-six C57BL/6 female mice (aged 6–8 weeks) were obtained in-house (University of Edinburgh) and infected with *P. chabaudi* clones AJ, AS, CR and ER from the Edinburgh Malaria reagent repository (University of Edinburgh). *Plasmodium chabaudi* was isolated between 1948 and 1974 from African thicket rats, *Thamnomys* spp., in Central Africa [29]. After cloning, the parasite genotypes have been cryopreserved and undergone regular transmission through mosquitoes to maintain their wild type phenotypes [30]. The four *P. chabaudi* genotypes used span the diversity of virulence (in terms of host anaemia and weight loss) reported from previous experiments [31–33]. We first describe the main experiment exploring genotype by environment interactions in asexual traits, and then describe a parallel experiment testing the effect of our environmental perturbation on the immune environment.

First, hosts were allocated to anaemia or control treatment groups. Anaemia was induced by administering 30 mg/kg phenylhydrazine (PHZ) in PBS carrier via intra-peritoneal injection and controls received only the carrier. PHZ causes premature lysis of RBCs through denaturation of haemoglobin, and, in consequence, induces an inflow of immature RBCs (reticulocytes) with an EPO-mediated feedback loop [34]. Inducing host anaemia with PHZ is a useful environmental perturbation because, unlike simply following infections through their natural course, the availability and age-structure of RBC resources can be manipulated independently of confounders resulting from the progression of infections (e.g., immune responses, parasite density). Four days post PHZ or carrier administration, all mice were infected with 5 × 10^6^ parasitized RBCs. Briefly, 7 hosts for each of the two treatment groups were infected with clones AJ, AS, CR or ER. All PHZ treatments and genotypes were randomized across 10 cages, with 8 cages each containing 6 mice and 2 cages each containing 4 mice. From day 1 to 5 post infection (PI), mice were monitored daily at 08.00 (GMT) by taking 2 μl of blood to quantify RBC density (Coulter Technologies) and making a thin blood smear to quantify asexual stages, the age structure of RBCs (measured as the proportion of RBCs that were reticulocytes, which are larger and stain bluer with Giemsa than mature RBCs), and the proportion of infected reticulocytes. At midnight on day 4 PI, a blood smear was made from each mouse to count the number of merozoites inside schizonts (burst size). From 08.00 on day 3 PI to 08.00 on day 5 PI, blood smears were made for each mouse every 8 h to assess the relative abundance of the developmental stages (staging them as either ring stage, trophozoite or schizont) to estimate the duration of the asexual cycle.

Next, a separate set of forty mice, host strain-, age-, and sex-matched, were injected with 30 mg/kg PHZ (n = 23) or PBS carrier (n = 17, control treatment) to test whether PHZ has an effect on the innate immune environment encountered by parasites on the day of infection. Specifically, the cytokines TNF-alpha and IFN-gamma, which kill asexual stages either directly or indirectly through the recruitment of macrophages [35], were assayed by ELISA (see Additional file 1 ‘Immune assays’ for further details).

### Quantification of traits

Asexual densities were calculated by multiplying the proportion of infected RBCs (estimated from blood smears) with the density of RBCs per ml of blood. Infections in PHZ-treated hosts reached peak on day 4 PI, thus to ensure that all infections were examined in their growth phase, densities from day 5 are not included in the replication rate analyses. Burst size was quantified for between 10 and 15 schizonts on each smear. Asexual cycle duration was estimated using a Bayesian-based computational model. Briefly, the model is based on a mathematical description of the phase distribution of parasites within their developmental cycle, classifying each as one of three stages (ring stage, trophozoite or schizont) at 8-h sampling points over 2 days. The joint likelihood of the model parameters was calculated using broad priors, which contributed little to the posterior because of the periodicity and the quantity of data. Finally, a Markov Chain Monte Carlo method was used to sample the posterior, which is highly suitable for sampling complex posteriors of high-dimensional, non-linear, multivariate dynamical systems (see Additional file 1 ‘Estimating cycle duration’ and ‘Inference of model parameters’ for further details). Invasion rates were calculated for reticulocytes and normocytes separately using equations 1 and 2 in [3] and reflect the probability that a parasite will invade an RBC and survive long enough to complete its cycle. These calculations account for the age structure and density of the available RBCs before and after invasion, as well as of the density of merozoites before invasion. Invasion rates were calculated only on day 4 PI because this was when the proportion of infected reticulocytes was high enough to return reliable estimates. To estimate the density of merozoites, the average burst size for each infection was multiplied by the density of ring stage parasites on day 3 PI. This model assumes that merozoite lifespan is equal across genotypes and PHZ combinations and that no death of uninfected RBCs takes place before or during parasite invasion, so that the total density of RBCs on day 4 PI is equal to the density of RBCs that the parasites were exposed to at the start of the invasion cycle.

### Statistical analysis

All statistical analyses were carried out using R (version 3.2.3). For burst size and asexual cycle duration, linear models (2-way-ANOVAs) were constructed relating the dependent variable of interest to genotype and PHZ treatment. The density of schizonts with a high burst size and invasion rates required square root transformation whereas reticulocyte proportion, asexual density and replication rate required log10 transformation. Maximum-likelihood based deletion tests were used to assess the effects of excluding each fixed effect. The dynamics (patterns observed during infections) of the proportion of RBCs that are reticulocytes, density of RBCs/ml blood, invasion rates, and asexual density/ml blood were analysed with linear mixed effect (LME) models, including PHZ treatment, genotype, and day PI, as fixed effects and with mouse identity as a random effect. The significance of fixed effects in LME models was determined by comparing the model including and the model excluding the fixed effect of interest using a likelihood ratio test. In minimized models where genotype or any interaction with genotype remained. Finally, a linear model was constructed to estimate the relative importance of traits (burst size, cycle duration, invasion rates, genotype) contributing to replication rate. For each statistical model, diagnostic plots of residuals were examined to assess whether linear modelling assumptions were satisfied [36, 37].

## Results

### Assumptions of the experimental design

**Fig. 1:**
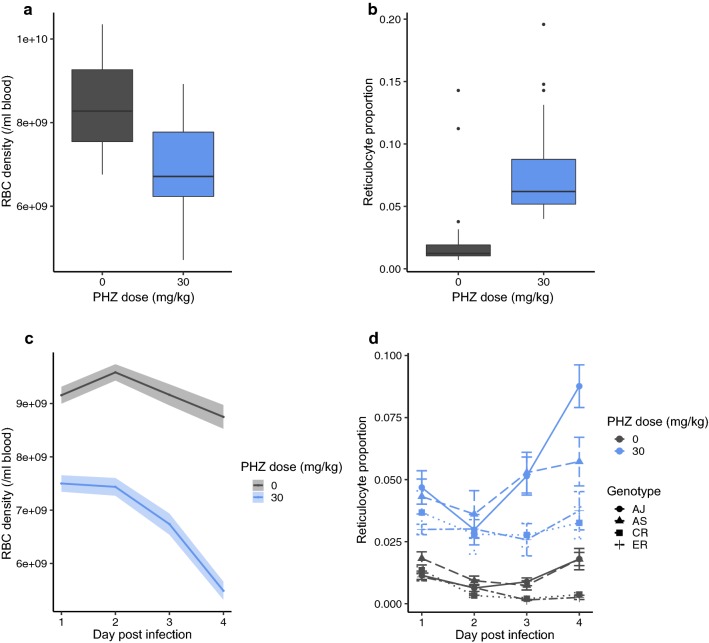
The difference in RBC environments between control (grey) and PHZ-treated (blue) hosts on the day before infection. For **a** total RBC density and **b** the proportions of RBC that are reticulocytes (box plot featured here and throughout present the median, interquartile range, and outliers). The difference in RBC environments between control and PHZ-treated hosts during infections for **c** RBC density (mean ± SEM) and **d** proportion of reticulocytes for each genotype (AJ: solid line + round point, AS: dashed line + triangle point, CR: dotted line + square point, ER: dot-dash line + cross point, mean ± SEM)

First, the experiment assumed parasites in PHZ-treated hosts enter and experience more anaemic within-host environments than parasites in control hosts. This requirement was met: all four genotypes of *P. chabaudi* experienced non-overlapping RBC environments between control and PHZ treatments (Fig. 1, Table 1). On the day before infection, regardless of which parasite genotype hosts were destined to be infected with, PHZ hosts exhibited significantly reduced RBC density and significantly increased proportion of RBCs that were reticulocytes (Fig. 1a, b). Specifically, mean total RBC density in control hosts was 8.42 billion cells (± 0.20) per ml, compared to 6.87 billion (± 0.22) in PHZ-treated hosts. Reticulocyte proportion was increased on average from 0.023 (± 0.006) to 0.077 (± 0.007) by PHZ. The RBC environments also remained significantly different between control and PHZ-treated hosts during the experiment (Fig. 1c, d). There was however, a significant genotype by day interaction for reticulocyte proportion dynamics, driven by a faster increase in AJ/AS infections compared to CR/ER infections, in PHZ-treated hosts.

**Table 1:** Statistical analysis of RBC density and reticulocyte proportion, both on the day before infection (− 1 PI) and during infections (dynamics)

	RBC density	
Day − 1 PI	G by PHZ	F(3,51) = 2.01, p = 0.125
	G	F(3,54) = 1.62, p = 0.196
	PHZ	F(1,52) = 27.72, p < 0.001*
Dynamics	G by PHZ by Day	χ^2^(3,18) = 3.67, p = 0.300
	G by PHZ	χ^2^(3,15) = 3.85, p = 0.278
	G by Day	χ^2^(6,15) = 8.11, p = 0.230
	Day by PHZ	χ^2^(1,15) = 22.64, p < 0.001*
	G	χ^2^(3,9) = 3.90, p = 0.272
	Proportion of reticulocytes	
Day − 1 PI	G by PHZ	F(3,51) = 1.88, p = 0.146
	G	F(3,54) = 0.74, p = 0.534
	PHZ	F(4,55) = 22.75, p < 0.001*
Dynamics	G by PHZ by Day	χ^2^(3,18) = 6.82, p = 0.078
	G by PHZ	χ^2^(3,15) = 4.42, p = 0.220
	Day by PHZ	χ^2^(4,15) = 25.07, p < 0.001*
	G by Day	χ^2^(6,15) = 27.36, p < 0.001*

Significant terms (p < 0.05), not eliminated from the model, are marked with an *. G stands for genotype, PHZ for phenylhydrazine treatment, day for day post-infection

Second, the assumption that PHZ caused a much stronger perturbation to RBC metrics than to immune responses was met. The standards in each ELISA assay yielded the expected concentrations of IFN-gamma and TNF-alpha. For IFN-gamma, one mouse was excluded because of poor repeatability between duplicates and two mice were positive but very low concentrations were detected (2.79 and 0.299 pg/ml) and belonged to a PHZ-treated group and a control group, respectively. For TNF-alpha, all mice showed good repeatability and were positive but very low concentrations were detected (15.6 and 20 pg/ml) and belonged to a PHZ-treated group and a control group, respectively. Note, the mice in which IFN-gamma was detected differed from those in which TNF-alpha was detected. Thus, parasites within PHZ or control groups all encountered very similar inflammatory cytokine environments upon and during infections.

### Asexual traits: density dynamics

**Fig. 2:**
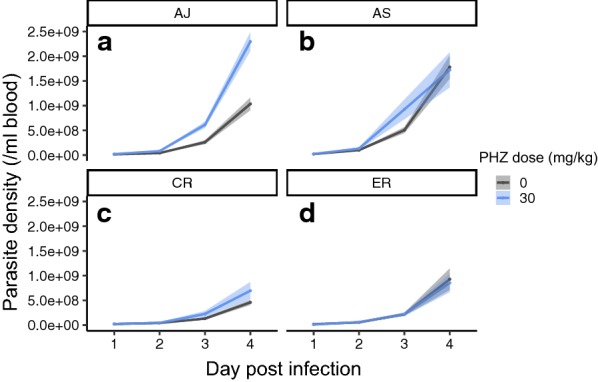
Mean (± SEM) daily asexual parasite density for each genotype in naïve control (grey) and PHZ-treated hosts (blue), for genotypes **a** AJ, **b** AS, **c** CR, and **d** ER

Asexual replication was significantly affected by whether parasites infected PHZ-treated or control hosts in a genotype-specific manner (genotype by PHZ interaction, Fig. 2, Table 2), with AJ exhibiting the greatest increase in densities in anaemic hosts.

**Table 2:** Statistical analysis of asexual density dynamics

Asexual density dynamics	
G by PHZ by day	χ^2^(9,34) = 7.97, p = 0.538
Day by PHZ	χ^2^(3,25) = 8.63, p = 0.035*
G by PHZ	χ^2^(3,25) = 8.91, p = 0.030*
G by day	χ^2^(9,25) = 59.16, p < 0.001*

Significant values (p < 0.05) are marked with a *. G stands for genotype, PHZ for phenylhydrazine treatment, day for day post-infection

### Asexual traits: burst size

**Fig. 3:**
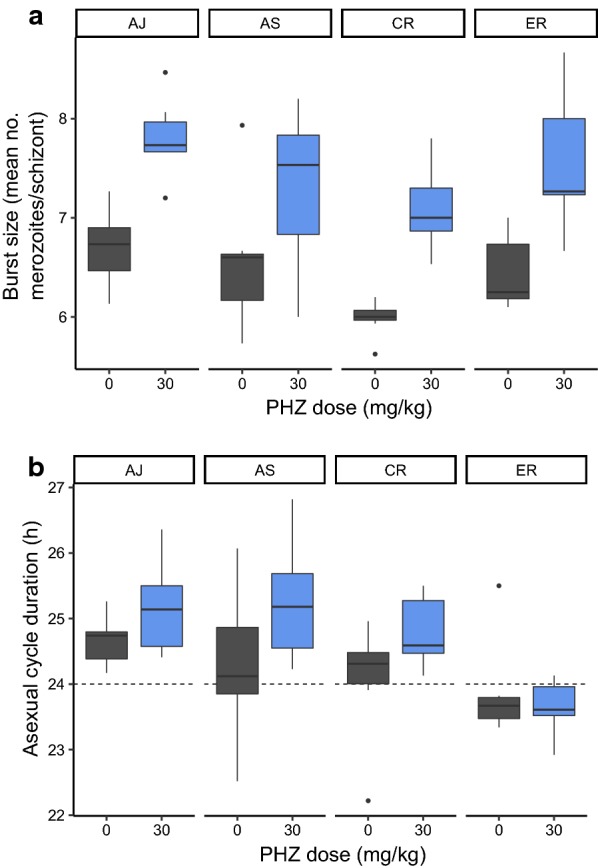
Burst size **a**, and asexual cycle duration **b**, for each genotype in naïve control (grey) and PHZ-treated hosts (blue). The dotted line in **b** marks a cycle duration of 24 h

Burst size could not be estimated for 3 mice because fewer than 10 mature schizonts were found on their blood smears. The effect of host anaemia on burst size did not differ across the genotypes (genotype by PHZ treatment F(3,51) = 0.45, p = 0.718, Table 3). However, burst size was significantly greater in PHZ-treated hosts (PHZ: F(1,55) = 46.25, p < 0.001, Fig. 3a), and lower in CR than the other genotypes (t value = − 2.57, p = 0.01). The minimal model explained 45.1% of the observed variance in burst sizes.

**Table 3:** Mean (± SEM) and the ratio of burst sizes across genotypes and treatments

	Genotype			
	AJ	AS	CR	ER
PHZ	7.81 (± 0.15)	7.29 (± 0.29)	7.10 (± 0.16)	7.58 (± 0.25)
Control	6.70 (± 0.15)	6.55 (± 0.26)	5.98 (± 0.70)	6.45 (± 0.14)
PHZ/control	1.17	1.11	1.19	1.17

PHZ stands for phenylhydrazine treatment

To investigate whether higher burst sizes can be explained by a higher proportion of reticulocytes, the proportion of infected reticulocytes observed on day 4 PI was compared to the density of schizonts with burst size > 6. There is a non-significant effect of reticulocyte proportion on the density of schizonts with high burst size (F(1,21) = 3.36, p = 0.08), with the proportion of infected reticulocytes in the blood only explaining 10.1% of the variance in the density of high burst-size schizonts. Overall, the proportion of infected reticulocytes in the blood of PHZ-treated hosts was on average only 1.1% (± 0.2), yet the total proportion of infected RBCs was 24.9% (± 0.03) by day 4 PI, suggesting it is unlikely that burst size is only determined by the age of the RBC a parasite resides in.

### Asexual traits: cycle duration

The duration of the asexual cycle is approximately 24 h in *P. chabaudi* [21, 38]. Asexual cycle duration varies depending on PHZ treatment (F(1,52) = 5.91, p = 0.019) and parasite genotype (F(3,54) = 6.22, p = 0.001), but with no significant interaction between the two (F(3,51) = 1.54, p = 0.22) (Fig. 3b). In general, the asexual cycle duration is at least 30 min longer in PHZ-treated hosts (especially for AJ, AS and CR) except ER which has a faster cycle than the others (Table 4). Overall, the minimal model explained 27.2% of the total variance in cycle duration.

**Table 4:** Mean (± SEM) and the difference (in hours:minutes) in asexual cycle duration across genotypes and treatments

Treatment	Genotypes			
	AJ	AS	CR	ER
PHZ	25:09 (± 00:16)	25:14 (± 00:20)	24:59 (± 00:12)	23:40 (± 00:10)
Control	24:39 (± 00:08)	24:19 (± 00:26)	24:04 (± 00:20)	23:52 (± 00:17)
(PHZ-control)	+ 00:30	+ 00:56	+ 00:45	− 00:12

PHZ stands for phenylhydrazine treatment

### Asexual traits: invasion rates

**Fig. 4:**
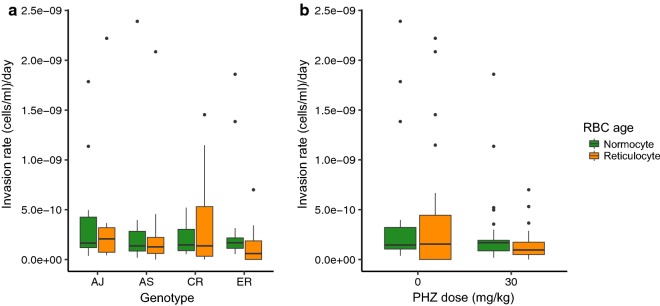
Invasion rates of normocytes (mature RBC, green) and reticulocytes (immature RBC, orange). For **a** all genotypes and **b** for different PHZ treatments (all genotypes combined). The rate reflects the probability of contact and successful invasion between uninfected cells and merozoites

The method used to calculate invasion rates can cause some estimates to be negative due to associated random measurement error between variables. In this data set, 5 out of 56 mice returned negative rates for both reticulocytes and normocytes and one mouse negative for only normocytes and were therefore excluded from this particular analysis. However, excluding or including these data points affected the results only quantitatively, not qualitatively. Invasion rates for both normocytes (Fig. 4a) and reticulocytes (Fig. 4b) varied but could not be explained by RBC age, PHZ treatment or parasite genotype, fitted either as main effects or within interactions (Table 5).

**Table 5:** Statistical analysis of invasion rates of differently aged RBCs

Invasion rates	
G by PHZ by Age	χ^2^(3,18) = 7.14, p = 0.068
G by PHZ	χ^2^(3,15) = 3.12, p = 0.374
G by Age	χ^2^(6,15) = 12.20, p = 0.058
Age by PHZ	χ^2^(7,15) = 13.32, p = 0.065
G	χ^2^(3,8) = 1.14, p = 0.768
PHZ	χ^2^(4,8) = 2.32, p = 0.677
Age	χ^2^(5,8) = 5.24, p = 0.387

Significant values are marked with an *. G stands for genotype, Age stands for age of RBC (normocyte or reticulocyte), and PHZ for phenylhydrazine treatment

### Relating asexual traits to replication rate

A model was constructed to ask whether the plasticity and genetic variation detected in asexual traits explain the different asexual dynamics exhibited by the genotypes in control and PHZ-treated hosts. Asexual dynamics were summarized as the change in density between day 1 to 4 PI (referred to as replication rate) and the model included main effects for PHZ, burst size and cycle duration, reticulocyte and normocyte invasion rates, and their interactions with parasite genotype (higher order interaction terms were omitted so as to guarantee sufficient degrees of freedom). The model simplified to include only main effects for genotype and burst size (Table 6), explaining 46.4% of the variance observed in replication rate. Asexual replication was positively associated with burst size for all genotypes, with AJ and AS generally achieving higher replication rates than CR and ER (see Fig. 2).

**Table 6:** Statistical analysis of whether variation in asexual traits associates with variation in replication rate

Full model	Replication rate ~ G + PHZ + burst size + cycle duration + normo.invasion + retic.invasion + G: PHZ + G: burst size + G: cycle duration + G: normo.invasion + G: retic.invasion
Minimal model	Replication rate ~ G + burst size
Non-significant terms removed from model	
G: cycle duration	F(3,28) = 0.29, p = 0.833
G: retic.invasion	F(6,31) = 0.40, p = 0.871
G: PHZ	F(9,34) = 0.63, p = 0.762
G: normo.invasion	F(12,37) = 0.68, p = 0.756
G: burst size	F(15,40) = 0.90, p = 0.576
PHZ	F(1,44) = 0.44, p = 0.509
Cycle duration	F(2,42) = 0.39, p = 0.680
Retic.invasion	F(3,43) = 0.45, p = 0.718
Normo.invasion	F(4,44) = 1.66, p = 0.178
Significant terms	
Burst size	F(5,45) = 2.50, p = 0.046*
Genotype	F(7,47) = 5.78, p < 0.001*

Significant values are marked with an *. G stands for genotype, ‘normo’ for normocyte, and ‘retic’ for reticulocyte, and PHZ for phenylhydrazine treatment

## Discussion

The experiment presented here finds that the impact of host anaemia differs between four genotypes of *P. chabaudi.* First, asexual density dynamics are altered in anaemic hosts in a genotype specific manner (G×E; the trait value of a particular genotype depends on its environment). Specifically, AJ exhibits a greater increase in replication in PHZ-treated hosts than AS, CR and ER, and AS has similar amounts of replication in both control and treatment groups. Second, CR exhibits a lower burst size (approx. 0.5 merozoites/schizonts) than the other genotypes and across all genotypes, burst size increases (approx. 1 merozoite/schizont) in PHZ-treated hosts. This increase may seem modest but when multiplied across millions of parasites within a cohort that replicate every day, a small increase can make a large contribution to within-host replication. *Plasmodium falciparum* genotypes also differ, on average, in burst size by 2 merozoites [39]. Third, ER exhibits a faster asexual cycle (approx. 1 h) than the other genotypes and across all genotypes, cycle duration slows (by approx. 35 min) in PHZ-treated hosts. This 2–4% divergence in cycle duration is similar to that reported for two *P. falciparum* genotypes (50 and 44 h, [39]). Fourth, in contrast to the other traits, variation in the invasion rates of differently aged RBCs is not associated with genotype or host anaemia, concluding *P. chabaudi* to be a generalist. Genotype-specific preferences for RBCs of different ages has been reported for *P. chabaudi* (e.g., reticulocyte preference for AS, [3]) and normocyte preference for AJ, [12] but these data are not from PHZ-treated hosts and differ qualitatively from those presented here. Fifth, plasticity in burst size and genotype identity are the only traits that make significant contributions to the variation observed in replication rates. However, > 50% of the variation in replication could not be explained, suggesting key roles for other traits such as sequestration behaviours and switching of surface antigens [40].

To what extent is the variation observed in asexual traits attributable to parasite control? Could the plasticity observed simply reflect how much the within-host environment directly affects the ability of parasites to achieve particular trait values? For example, are parasites programmed to achieve the same burst size regardless of their environment, but unable to achieve this trait value in control hosts due to a lack of resources? If so, the increase in burst size in PHZ-treated hosts may simply be the outcome of an external constraint being lifted rather than active decision by the parasite to modulate burst size. In support of the former, conversion rate is increased in PHZ-treated hosts, which suggests the parasite is better resourced in PHZ-treated hosts [33]. However, in support of latter, parasites can control burst size [10]. Alternatively, cycle duration might be extended in anaemic hosts simply because blood with a low RBC density may not generate sufficient friction to facilitate schizonts bursting on time [38]. In reality, parasite traits are likely shaped both by parasites adjusting their strategies and the constraints imposed (or opportunities provided) by the prevailing within-host conditions.

Further complications arise if multiple traits are correlated. For example, achieving a high burst size may require a long asexual cycle to garner sufficient resources and construct merozoites. If so, parasites might benefit from actively increasing burst size but how much they can alter this trait is constrained by the need keep the cycle duration as close to 24 h as possible. However, given that *Plasmodium berghei* typically produces 12–18 merozoites per schizont (i.e., twice as many as *P. chabaudi*) from a shorter cycle [29] and here, ER alters burst size independently of cycle duration, burst size is not inevitably linked to cycle duration. Furthermore, the genotypes used here (AJ, AS, CR, ER) also adopt different conversion rates in PHZ-treated hosts [33]. Because conversion rate trades off against asexual density, a high converting genotype could suffer low asexual densities as a consequence [6]. This is unlikely to be the case here because the genotype groupings for the effects of PHZ on asexual densities and conversion rates differ: asexual densities of AJ are the most affected by PHZ (Fig. 3) but, out of all 4 genotypes, AS responds with the biggest increase in conversion and ER with the smallest increase (Fig. 3 from [33]).

The same difficulties in ascertaining the extent to which plasticity in asexual traits is controlled by the parasite also apply to interpreting genetic variation. For example, can the differences between genotypes in PHZ-treated hosts be explained simply by them exacerbating PHZ-induced anaemia to different extents? The dynamics of RBC density (normocytes plus reticulocytes, Fig. 1c) did not vary in a genotypic-specific manner during infections (of both PHZ-treated and control hosts) suggesting the differences in replication rate are independent of RBC densities. Similar logic can be applied to an influence of reticulocyte proportion on genotype differences in control hosts. However, reticulocyte proportion dynamics did vary between the genotypes in PHZ-treated hosts and in this group, reticulocyte proportion correlates closely with asexual density on day 4 (F(4,27) = 6.45, p = 0.001; adj. R^2^ = 0.38) but genotype does not (F(3,26) = 2.02, p = 0.14). Parasites achieving higher densities in anaemic hosts simply because they perform better in reticulocytes is not consistent with two observations. First, that plasticity in burst size is not explained by the age of RBC a parasite infects. Second, that parasites do not differ in their ability to invade normocytes or reticulocytes. Further work should examine whether the different reticulocyte proportion dynamics are due to chance variation between hosts, resulting from how different genotypes influence erythropoiesis or the development of innate immune responses, for example RBC-age specific filtering by the spleen. Using different doses of PHZ to induce different levels of anaemia could also discriminate between genetically encoded differences in replication rate in anaemic hosts from the consequences of abundant resources.

G×E interactions in fitness-related traits are key contributors to evolution [23]. This is because environmental change can expose genetic variation and allow natural selection to change gene frequencies in the population. For instance, if all genotypes exhibit similar values for a particular fitness trait in control infections, then natural selection operating in this environment cannot sort between genotypes [23, 24]. In contrast, if the trait values of some genotypes increase (suggesting higher fitness) in anaemic hosts but the trait values for other genotypes remain unchanged or decrease, selection pressures resulting from infecting anaemic hosts are able to favour the genotypes with higher fitness, and they rise in frequency in the population. Such a phenomena may operate on replication rate because the rank order of genotypes differs between control and anaemic hosts (e.g., AS reaches the highest density in control hosts but is lower than AJ in PHZ-treated hosts). Extending this to natural infections suggests that interventions affecting the prevalence or degree of anaemia may facilitate a change in parasite gene frequencies. Furthermore, a host population with variable levels of anaemia may maintain genetic variation in the parasite population.

## Conclusion

Explaining variation in parasite traits is inherently challenging because quantitative and qualitative characteristics of an infection are the result of both host and parasite effects [11]. Additional complexity is introduced if parasite traits are phenotypically plastic and vary according to within-host-conditions, in manners that differ between genotypes (G×E). Given that asexual replication is responsible for host exploitation and virulence, it is paramount to improve approaches for quantification of other asexual traits such as sequestration behaviours and switching between variable surface antigens [17]. Furthermore, the present study focuses on using anaemia as an environmental perturbation but plasticity and genetic variation in asexual traits is likely in response to a range of within-host pressures, including co-infection, immunity and drug treatment. Predicting treatment success and preventing evolution in response to anti-malarial interventions could be facilitated by better understanding the drivers of variation in both asexual and sexual traits of parasites and the correlations between them. For example, if different ligand-receptor interactions used by different *P. falciparum* genotypes drive variation in both invasion phenotypes and immune selection [41], this should be accounted for in vaccine development.

## Additional file

**Additional file 1.**

 Detailed methods for immune assays, estimating cycle duration [42] and inference of model parameters [43–46].

## Data Availability

The datasets used and/or analysed during the current study are available from the corresponding author on reasonable request.

## Abbreviations

RBC: red blood cell
E: environment effects
G: genotype effects
G×E: genotype by environment effects
PHZ: phenylhydrazine
PI: post infection
PBS: phosphate-buffered saline
EOP: erythropoietin
