# Supplementary material for: Plasticity and genetic variation in traits underpinning asexual replication of the rodent malaria parasite, Plasmodium chabaudi
Source: Malar J. 2019 Jul 1;18:222. doi: 10.1186/s12936-019-2857-0 (PMC6604315; doi:10.1186/s12936-019-2857-0)
Supplement: Supplementary file 1 — Additional file 1. [file 12936_2019_2857_MOESM1_ESM.docx]

**Additional information**

**Immune assays**

Blood was obtained by cardiac puncture, centrifuged twice for 10 minutes at 60,000 rpm, resulting in about 200 ul of plasma. Plasma was shock-frozen on dry ice and stored at -80°C. An ELISA was used to detect the levels of TNF and INF-gamma following the manufacturer's protocol (Invitrogen). For INF-gamma, a range of 7 standards was used (included in the kit) ranging from 1.7 pg/ml to 300 pg/ml (as recommended by the manufacturer). For TNF, a range of 9 standards was used, ranging from 7.8 pg/ml to 2,000 pg/ml (adding one additional concentration on each end of the range recommended in the kit). All standards and all samples were run in duplicates, and for each mouse the mean of readings at an absorbance of 450 nm was taken, as recommended by the manufacturer. Samples with poor repeatability between duplicates were excluded. ELISA software (elisaanalysis.com) was used to fit standard curves and infer concentrations present in samples. Each plate contained multiple negative controls and non-specific binding assays (chromogen blanks). Samples that gave a reading below that of a negative control were excluded.

**Estimating cycle duration**

The model is based on a mathematical description of the phase distribution of parasites within their developmental cycle $f(\phi,t)$ where $\phi\in[0,1)$ is the phase of the developmental cycle and $t$ is time. Thus $f(\phi,t)$ tells us where the bulk of the parasites are in their developmental cycle at a given time $t$. By convention, parasites that have just invaded an erythrocyte have phase $\phi=0$ and parasites that have just burst from an erythrocyte have phase $\phi=1$. We assume that, as parasites develop through their asexual cycle, their degree of synchronicity amongst themselves remains constant. This means that the shape and width of the phase distribution remains constant with time, only its location changes with time.

Let $S=(\text{ring, troph, schizont})$ be the sequence of developmental stages. Let $\tau_{s}$ be the mean duration of stage $s$ in hours. These add up to the mean cycle length $L.$ We do not explicitly model merozoites and their invasion of erythrocytes. Instead we assume that each bursting schizont immediately gives rise to a number $\lambda$, of newly infected erythrocytes. This is a reasonable assumption given that merozoites invade erythrocytes in a matter of minutes [42] compared to the daily time-scale of parasite development within erythrocytes. Bursting and re-invasion determines the boundary conditions of the phase distribution, that is, $f\left( 0,t \right)=\lambda f\left( 1,t \right).$

The change of the phase distribution over time can be modelled by the advection equation

$$\frac{\partial f}{\partial t}+\frac{1}{L}\frac{\partial f}{\partial t}=0$$

Rather than numerically solving this equation, which is non-trivial, we discretise the parasite phase distribution and solve the resulting recurrence equations. These are simpler and faster to solve and do not introduce numerical diffusion.

Let $\Delta t$ be the simulation time-step. The phase distribution is discretised into $N$ equally spaced compartments, such that $N=\frac{L}{\Delta t}$. Let $x_{j}(t)$ for $j=1,\ldots$,N be the frequency of parasites in compartment $j$ at time $t$. For the case of a stable phase distribution, the time evolution of the phase distribution is given by the following system of recurrence equations

$$x_{1}\left( t+\Delta t \right)=\lambda x_{N}\left( t \right)$$

$$x_{j}\left( t+\Delta t \right)=x_{j-1}\left( t \right) \text{for }j=2,\ldots,N$$

Setting the timestep $\Delta t$, is a trade-off between computational time and precision of the solution. In trial runs we found that $\Delta t=0.5$ h gave reasonable simulations times and precise solutions.

The shape of the phase distribution is unknown. Experimentation with various distributions suggests, however, that stage dynamics are insensitive to its precise form. A reasonable guess is the Von Mises distribution. This is a close approximation to a wrapped normal distribution with location (mean) $\phi_{0}\in\left[ 0,1 \right)$and precision $\kappa\in\left[ 0,\infty\right).$ It has the form $g\left( \phi;\phi_{0},\kappa\right)\propto e^{\kappa\cos(2\pi\left( \phi-\phi_{0} \right)}$. The Von Mises distribution is such that $g\left( 0 \right)=g(1)$. However, in a growing population where each bursting schizont gives rise to $\lambda$ new ring stages, we require $f\left( 0,t \right)=\lambda f\left( 1,t \right)$. We can achieve this by multiplying the Von Mises distribution by the factor $e^{-\phi\ln\lambda}$:

$$f\left( \phi,0 \right)=Ae^{-\phi\ln\lambda}e^{\kappa\cos(2\pi\left( \phi-\phi_{0} \right)}$$

(3)

where $A$ is a scaling factor of no interest as we work with proportions rather than with frequency. The parameter $\kappa$ can be thought of as a measure of the parasites’ degree of synchrony amongst themselves. If $\kappa$ is zero, the parasites are completely desynchronised; parasites are to be found equally at all phases of the cycle. As$\kappa$ increases parasites become more synchronised, until as $\kappa\to\infty$ the parasites are completely synchronised; they are all at the same phase of the cycle at any given time.

The initial parasite frequency in each compartment is found by integrating the initial phase distribution $f(\phi,0),$ between the appropriate limits:

$$x_{j}\left( 0 \right)=\int_{\frac{j-1}{N}}^{\frac{j}{N}} f\left( \phi,0 \right)d\phi\text{for} j=1,\ldots,N$$

Finally we assume that a proportion $\sigma$, of schizonts sequester in deep tissues making them unavailable for sampling.

## Inference of model parameters

Let $\theta(\tau_{\text{ring}},\tau_{\text{troph}},\tau_{\text{schizont}},\lambda,\phi_{0},\kappa,\sigma)$ be the vector of model parameters to be estimated. Simulating the model with a particular set of parameter values generates time-series of the probabilities of observing a parasite in one of the three developmental stages. Let $p_{\text{ring}}\left( \theta,t \right),$ $p_{\text{troph}}\left( \theta,t \right)$and $p_{\text{schizont}}\left( \theta,t \right)$be these probabilities.

Let $M$ be the number of sampling time points. At each time point in the experiment the observed proportions of the three stages are recorded. Let these proportions be $P_{\text{ring}}\left( t_{i} \right),$ $P_{\text{troph}}\left( t_{i} \right)$and $P_{\text{schizont}}\left( t_{i} \right)$ at time point $t_{i}$ for $i=1,\ldots,M$. The observed proportions are assumed to be normally distributed with mean $p_{s}(\theta,t_{i})$ and standard deviation $p_{s}(\theta,t_{i})(1-p_{s}\left( \theta,t_{i} \right))$ for $s\in S$. The joint likelihood $\mathcal{L}$, of the model parameters given the data up to a constant of proportionality, is

$$\mathcal{L}\left( \theta\right)\propto\prod_{i=1}^{M} \prod_{s\in S} \Pr(P_{s}\left( t_{i} \right) | p_{s}(\theta,t_{i}) )$$

Priors were broad and contributed little to the posterior due to the periodicity and quantity of data. We set $L\sim\text{Normal}(24, 1)$, $\phi_{0}\sim\text{Uniform}(0, 1),$ $\kappa\sim\text{Gamma}(10, 1)$, $\tau_{\text{ring}}\sim\text{Normal}_{\text{Trunc}}(10, 4^{2})$ and $\tau_{\text{troph}}\sim\text{Normal}_{\text{Trunc}}(10, 4^{2})$. Growth rate $\lambda$ was fixed at 2 as sampling occurred during the exponential growth phase.

We use an adaptive, population based, Markov chain Monte Carlo method with power posteriors to sample the posterior [43-45]. This method is ideal for sampling complex posteriors of high-dimensional, nonlinear, multivariate dynamical systems. The Markov chain has a burn-in of $5\times{10}^{5}$ samples. Inferences are based on $4\times{10}^{6}$ samples thinned to 4,000 samples to reduce auto-correlation. Gelman-Rubin statistics [46] and plots of the Markov chains were examined to ensure satisfactory mixing and convergence.
